# The Antigen Presenting Potential of CD21^low^ B Cells

**DOI:** 10.3389/fimmu.2020.535784

**Published:** 2020-10-21

**Authors:** Marlene E. Reincke, Kathryn J. Payne, Ina Harder, Valentina Strohmeier, Reinhard E. Voll, Klaus Warnatz, Baerbel Keller

**Affiliations:** ^1^ Department of Rheumatology and Clinical Immunology, Faculty of Medicine, University of Freiburg, Freiburg, Germany; ^2^ Center for Chronic Immunodeﬁciency, Faculty of Medicine, University of Freiburg, Freiburg, Germany; ^3^ Faculty of Biology, University of Freiburg, Freiburg, Germany

**Keywords:** CD21^low^ B cells, antigen-presenting cells, autoimmunity, co-stimulation, T cell

## Abstract

Human CD21^low^ B cells are expanded in autoimmune (AI) diseases and display a unique phenotype with high expression of co-stimulatory molecules, compatible with a potential role as antigen-presenting cells (APCs). Thus, we addressed the co-stimulatory capacity of naïve-like, IgM-memory, switched memory and CD27^neg^IgD^neg^ memory CD21^low^ B cells in allogenic co-cultures with CD4 T cells. CD21^low^ B cells of patients with AI disorders expressed high levels of not only CD86, CD80, and HLA-DR (memory B cells) but also PD-L1 *ex vivo* and efficiently co-stimulated CD4 T cells of healthy donors (HD), as measured by upregulation of CD25, CD69, inducible co-stimulator (ICOS), and programmed cell death protein 1 (PD-1) and induction of cytokines. While the co-stimulatory capacity of the different CD21^low^ B-cell populations was over all comparable to CD21^pos^ counterparts of patients and HD, especially switched memory CD21^low^ B cells lacked the increased capacity of CD21^pos^ switched memory B-cells to induce high expression of ICOS, IL-2, IL-10, and IFN-γ. Acknowledging the limitation of the *in vitro* setting, CD21^low^ B cells do not seem to preferentially support a specific T_h_ effector response. In summary, our data implies that CD21^low^ B cells of patients with AI diseases can become competent APCs and may, when enriched for autoreactive B-cell receptors (BCR), potentially contribute to AI reactions as cognate interaction partners of autoreactive T cells at sites of inflammation.

## Introduction

Beside the production of antibodies, B cells contribute crucially to the adaptive immune response as potent antigen-presenting cells (APCs) (1). After endocytosis *via* the B-cell receptor (BCR) and processing of an antigen, B cells present antigenic peptides by MHC II molecules to CD4 T cells. This cognate interaction is strongly enhanced by activation-induced expression of co-stimulatory molecules like CD80 and CD86 on the surface of the antigen-presenting B cell binding to CD28 and other molecules involved in T-cell–B-cell (T-B) interaction. Therefore, memory B cells already expressing higher levels of these molecules are better APCs (2, 3) and in the absence of both molecules the activation of T cells is strongly impaired (4).

In the last 20 years, an accumulation of a circulating CD21^low^ B-cell population has been described in the context of different disease entities associated with chronic immune stimulation as in viral [human immunodeficiency virus (HIV) (5) or hepatitis C virus (HCV) (6)] or parasite infection [malaria (7)], in patients with immune dysregulation in common variable immunodeficiency (CVID) (8, 9), in graft versus host disease (10), or in autoimmune (AI) disorders like systemic lupus erythematosus (SLE) (11) or rheumatoid arthritis (RA) (12). We recently demonstrated that an accumulation of CD21^low^ B cells was most frequently observed in SLE patients, followed by RA and primary Sjögren Syndrome (pSS) but less frequently in undifferentiated or mixed connective tissue disease (UCTD/MCTD) or systemic sclerosis (SSc) (13). The different naïve-like (IgD^pos^CD27^neg^) or memory (IgD^pos^CD27^pos^, IgD^neg^CD27^pos^, and IgD^neg^CD27^neg^) CD21^low^ B-cell populations display a common core phenotype and share altered signaling characteristics independent of the underlying autoimmune disorder (13), some of which have been previously described as activated naïve (14), atypical memory (15), or tissue-like-memory (16) B cells. Their high expression of activation markers and co-stimulatory ligands for T-cell help, such as CD80 and CD86 (12, 16–19), distinctly discriminates them from their CD21^pos^ counterparts as potentially potent APCs for T cells (20, 21). This is of special interest since these CD21^low^ B-cell populations contain increased proportions of antigen-specific clones in chronic infection (22, 23) and of autoreactive clones in AI diseases (12, 14, 17, 24). Furthermore, several studies indicated that B cells play a prominent role as APCs in the induction of autoimmunity [ (25, 26) and reviewed in (1)]. Thus, given the conceivable pathological impact of increased co-stimulatory capacities, we addressed the co-stimulatory potential of the different CD21^low^ B-cell subsets in the context of AI disease in an allogenic superantigen-driven lymphocyte reaction.

## Material and Methods

### Patients

All experiments were performed with ethical approval by local authorities (Freiburg 239/1999 and 121/11 and Freiburg 66/13) according to the declaration of Helsinki. All patients and healthy donors (HD) had signed the informed consent.

In total 29 patients were included in the study at the outpatient clinic of the Department of Rheumatology and Clinical Immunology, University Medical Center Freiburg. 13 patients were diagnosed with RA, 8 with SLE, 2 with psoriatic arthritis, and 1 each with eosinophilic granulomatosis with polyangiitis (EGPA), pSS, antisynthetase-syndrome, sarcoidosis, spondyloarthropathy, and antiphospholipid syndrome. The patient cohort included 7 male and 22 female individuals with a mean age of 61.4 years (SD: +/- 13.8 years). Patient and HD cohort were not matched for age and gender. Disease duration ranged from 2 to 56 years. C-reactive protein (CRP) of patients ranged from <3 to 25.5 mg/l, with one outlier of 96.2 mg/l. Disease Activity Score 28 (DAS-28) of patients with RA ranged from 1.8 to 4, including 10 patients in remission (DAS-28 < 2.6) and 2 patients with low disease activity (DAS-28 < 3.2). Systemic lupus erythematosus disease activity index (SLEDAI) of patients with SLE ranged from 0 to 4, with the exception of one patient with active disease (SLEDAI of 8). Treatment of patients included no more than 8.75 mg prednisolone/day. Some patients were currently treated with monoclonal antibodies against TNF-α (n = 4) or IL-6R (n = 1). Other immunosuppressive therapies included methotrexate, hydroxychloroquine, leflunomide, azathioprine, and mycophenolate-mofetil. Patients’ characteristics and percentage of CD21^low^ B cells are summarized in **Table 1**.

**Table 1:** Patients’ characteristics.

AI patients	Disease	CD21^low^ B cells in %
#1	RA	17.8%
#2	RA	17.9%
#3	RA	12.4%
#4	pSS	10.2%
#5	SLE	7.2%
#6	RA	15.2%
#7	EGPA	14.0%
#8	RA	14.2%
#9	SLE	12.2%
#10	Antisynthetase-Syndrome	27.4%
#11	RA	16.7%
#12	SLE	10.6%
#13	RA	6.3%
#14	RA	1.6%
#15	Psoriatic arthritis	2.5%
#16	SLE	4.4%
#17	Sarcoidosis	1.4%
#18	SLE	1.3%
#19	RA	13.0%
#20	SLE	2.7%
#21	RA	22.7%
#22	RA	7.7%
#23	Psoriatic arthritis	3.0%
#24	Spondyloarthropathy	4.5%
#25	SLE	5.0%
#26	SLE	23.3%
#27	RA	14.4%
#28	RA	11.9%
#29	Antiphospholipid syndrome	15.5%

### Antibodies Used in This Study

Anti-CD19 APC-Cy7, anti-CD38 PerCp-Cy5.5, anti-CD4 PE-Cy7, anti-CD25 PerCP-Cy5.5, anti-CD86 APC, anti-CD80 BV421, anti-CD45RA BV605, anti-ICOS PE, anti-CD21 PE-Cy7, anti-CD80 BV421, anti-CD27 PerCp-Cy5.5, anti-PD-1 APC, anti-CD45RA APC-Cy7, anti-HLA-DR BV605, anti-CD11c AF700, anti-CD86 BV711, anti-CD95 BV650, anti-IgD BV786, anti-FCRL5 APC, anti-CXCR3 BV421, anti-CCR6 BV605, anti-CD19 BV650, anti-CD19 BV605, anti-PD-L1 BV421, anti-PD-L2 PE, anti-ICOS-L PE, anti-OX40L PE, anti-IL-10 PE, anti-IL-10 APC, anti-TNF-α APC-Cy7 (all obtained from BioLegend); anti-IgD FITC and anti-IgD PE (both from Southern Biotech); anti-CD27 PE (Dako); and anti-CD21 PE-Cy7, anti-CD69 FITC, anti-HLA-DR FITC, anti-CD27 BV605, anti-CD40 FITC, anti-CD40L PE, anti-ICAM-1 PE, anti-CXCR5 BUV395 and anti-CD27 BUV395 (all from BD Biosciences).

### Cell Staining and Isolation

**Figure 1:**
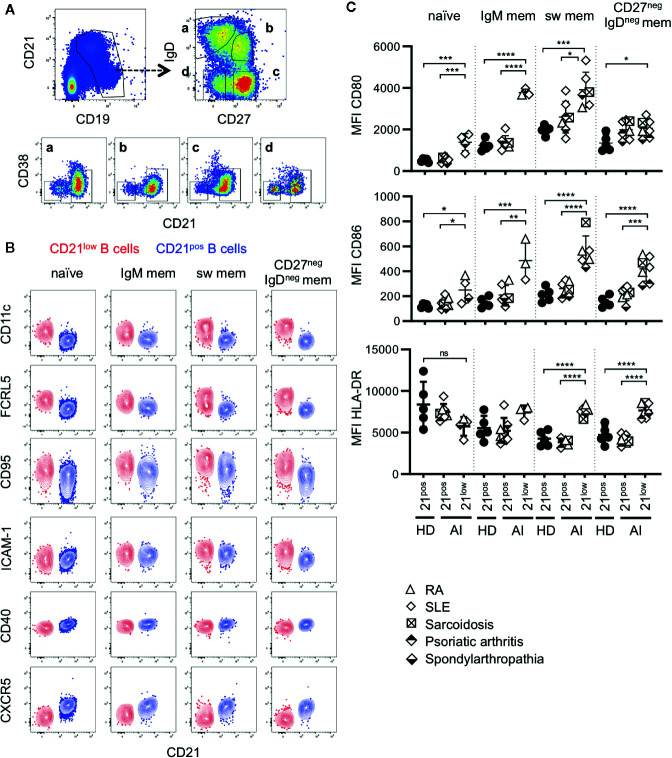
**(A)** General gating strategy for different CD21^low^ and CD21^pos^ B-cell subsets. CD19^pos^ B cells delineated by CD19 and CD21 were separated into naïve (a), IgM memory (IgM mem) (b), switched memory (sw mem) (c), and CD27^neg^IgD^neg^ memory (CD27^neg^IgD^neg^ mem) B cells (d) by expression of IgD and CD27. Subsequent gating on CD21^pos^CD38^low-intermediate^ and CD21^low^CD38^low^ B cells allowed the differentiation of CD21^pos^ and CD21^low^ B cells, respectively. **(B)** FACS plots showing CD21 and CD11c, FCRL5, CD95, ICAM-1, CD40 and CXCR5 in naive, IgM mem, sw mem and CD27^neg^IgD^neg^ mem B cells in one representative AI patient. **(C)** Graphs show the MFI of CD80, CD86 and HLA-DR in CD21^pos^ B-cell subpopulations of healthy donors (HD) and CD21^pos^ and CD21^low^ B-cell subsets of patients with autoimmune (AI) diseases in naïve, IgM mem, sw mem and CD27^neg^IgD^neg^ mem B cells. Statistical differences were considered significant at *p < 0.05, **p < 0.01, ***p < 0.001, and ****p < 0.0001. RA, SLE, sarcoidosis, psoriatic arthritis, and spondyloarthropathy were marked by different symbols as indicated in the legend. ns, not significant.

Peripheral blood mononuclear cells (PBMCs) were isolated from EDTA blood by Ficoll density centrifugation following standard protocols. Surface staining on T-B-co-cultures, fresh or frozen PBMCs was performed with optimal amounts of the respective antibodies at 4° C for 15 min. Sorting of B-cell subpopulations of patients and HD was performed on freshly isolated PBMCs within 4 h after blood withdrawal. As indicated in **Figure 1A** CD19^pos^ B cells were divided into naïve (CD27^neg^IgD^pos^), IgM memory (CD27^pos^IgD^pos^), switched memory (CD27^pos^IgD^neg^), and CD27^neg^IgD^neg^ memory B cells. CD21^pos^ and CD21^low^ subsets were distinguished from each of these subpopulations by subsequent gating on CD21^low^CD38^low^ and CD21^pos^CD38^dim^ B cells. B-cell sorting of four to six of the eight potential subpopulations within each sample was conducted with the MoFlo Astrios (Beckman Coulter) or the Aria Cellsorter (BD). The sorted subsets were chosen according to the proportion of the respective populations. For information on sorted subpopulations please refer to **Supplementary Table 1**.

CD4 T cells were isolated from HD by negative selection using the Magnetic Cell-Sorting (MACS) CD4+ T-cell isolation kit of Miltenyi Biotec following the manufacturer’s instructions. The purity of isolated T cells was over 95% as determined by flow cytometry. For each experiment, B-cell subpopulations of one patient and one HD (B-cell HD) were matched with CD4 T cells of another HD (T-cell HD). DAPI was added before acquisition of data for exclusion of dead cells when necessary. To determine intracellular cytokines, T and B cells were co-cultured as described below. 10 µg/ml BFA (Sigma-Aldrich) was added 6 h prior to harvest. Cells were fixed and permeabilized with Cytofix/Cytoperm and Perm/Wash Buffer (both BD Biosciences) following the manufacturer’s instructions, and samples were stained with the respective antibodies at optimal concentrations. Flow cytometric data was acquired at the LSR Fortessa (BD Biosciences).

### Mixed Lymphocyte Culture and Cell Activation

For T-B-cell co-cultures, 12,500 sorted B cells of patients and HD were cultivated either with or without 100 ng/ml of *Staphylococcus* enterotoxin B (SEB) (Sigma-Aldrich) in IMDM (Life technologies GmbH) containing 10% FCS (Biochrom) and 100 U/ml of penicillin/streptomycin (Life technologies GmbH). 18 h later allogenic CD4 T cells of a third-party T-cell HD (37,500 cells/well) were added and co-cultured for 24 h. After 24 h, supernatants were collected and stored at -80°C for subsequent analysis. As a positive control for activation, B cells were cultured with 2.5 µg/ml CpG alone for 42 h. Cells were washed and stained for flow cytometric analysis.

### Measurement of Cytokine Production

Cytokines were measured from the supernatants of co-cultures and SEB-stimulated T-cell cultures as described above. Measurements were conducted by a bead-based immunoassay (LEGENDplex™ Multi-Analyte Flow Assay Kit, Human T_h_ Cytokine Mix and Match Subpanel, BioLegend) according to manufacturer’s instructions and analyzed using the LSR Fortessa and the LEGENDplex software.

### Statistical Analysis

The data was analyzed using GraphPad Prism 7.0. Data were tested for normal distribution. For comparison of three groups, statistical differences were determined by parametric one-way ANOVA or non-parametric Kruskal-Wallis testing (both unpaired) using Tukey’s or Dunn’s post-hoc test for multiple comparison. In case of two groups, unpaired t-test was applied. Differences were considered statistically significant at *p < 0.05, **p < 0.01, ***p < 0.001, or ****p < 0.0001. Data are shown as means with standard deviation (SD).

## Results

### CD21^low^ B Cells Express High Levels of Co-Stimulatory Molecules *Ex Vivo*

CD21^low^ B cells defined by low expression of CD21 and CD38 constitute a common B-cell subset, comprising four stages of B-cell differentiation of naïve-like, non-switched, switched and CD27^neg^IgD^neg^ memory in analogy to their CD21^pos^ counterparts (**Figure 1A**) (13). According to literature (6, 12, 13, 15–19, 24, 27, 28), all four subsets express common markers of CD21^low^ B cells as increased expression of CD11c, FCRL5, CD95 and ICAM-1, reduced expression of CD40, and are negative for CXCR5 (**Figure 1B** and **Supplementary Figure 1A**). Concerning the co-stimulatory potential of CD21^low^ B cells, flow cytometric analysis revealed that the expression of the co-stimulatory molecules CD80 and CD86 was increased on all CD21^low^ B-cell populations when compared to the respective CD21^pos^ population of HD (**Figure 1C**). Comparing between the four CD21^low^ B-cell populations, CD80 and CD86 were highest on the CD27^pos^ B-cell compartments, and the lowest expression was observed on naïve-like CD21^low^ B cells, which was similar to the pattern observed on CD21^pos^ B-cell populations. In contrast, HLA-DR expression was higher on naïve vs. memory CD21^pos^ B cells, while this relation was reversed among CD21^low^ B-cell populations, displaying significantly increased expression on both CD21^low^ switched memory B-cell compartments when compared to the respective CD21^pos^ counterparts (**Figure 1C**). Expression of the co-stimulatory molecules ICOS-L and OX40-L was equally expressed on CD21^low^ and respective CD21^pos^ B-cell subsets except for significantly increased expression of ICOS-L on CD27^neg^IgD^neg^ memory CD21^low^ B cells (**Supplementary Figure 1B**). Additionally, we observed an increased expression of inhibitory programmed cell death ligand 1 (PD-L1) on CD21^low^ B cells reaching significance for CD27^neg^ B-cell subsets and comparable or slightly increased levels of PD-L2, which was only significant for switched memory CD21^low^ B cells (**Supplementary Figure 1C**). Thus, all CD21^low^ B-cell subsets share a distinct, activated phenotype prone for co-stimulation of T cells at sites of inflammation.

### Induction of Co-Stimulatory Molecules On CD21^low^ B Cells *In Vitro*

**Figure 2:**
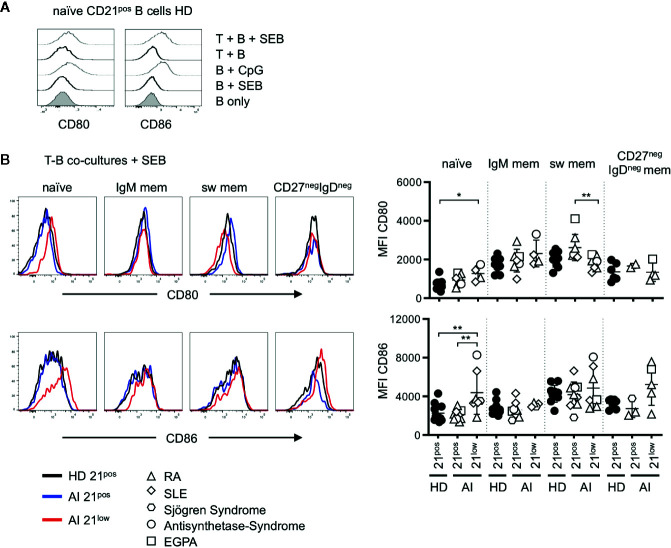
**(A)** Representative histogram overlays for the expression of CD80 and CD86 on naïve B cells of HD unstimulated, stimulated with SEB, CpG and in T-B co-cultures without or in the presence of SEB. **(B)** Histogram overlays for CD80 (upper line) and CD86 (bottom line) of naïve, IgM mem, switched mem and CD27^neg^IgD^neg^ mem CD21^pos^ B cells of one representative HD (black solid line) and CD21^pos^ (blue solid line) and CD21^low^ B cells (red solid line) of one representative AI patient in *Staphylococcus* enterotoxin B (SEB-) stimulated co-cultures with allogenic T cells and statistical analysis thereof. Differences were considered significant at *p < 0.05 and **p < 0.01. The different diseases were marked by different symbols as indicated in the legend.

To evaluate the co-stimulatory capacity, the four different subsets of CD21^low^ B cells and the respective CD21^pos^ populations of patients with AI disorders and HD were co-cultured with allogenic CD4 T cells of third-party healthy individuals in the presence of SEB. In the absence of T cells, CD80 and CD86 expression on SEB-stimulated B cells was comparable to unstimulated B cells (**Figure 2A**). Similarly, co-cultures of B cells and allogenic T cells in the absence of SEB did not induce an increased expression. However, after addition of SEB the expression of CD80 and CD86 was comparable to B-cell activation with CpG (**Figure 2A**).

Unlike ex vivo, in T-B co-cultures without SEB (“unstimulated co-cultures”) CD80 and CD86 expression on CD21^low^ B cells were only higher on naïve-like and non-switched memory B cells, but not anymore on switched memory B cells when compared to their CD21^pos^ counterparts (**Supplementary Figure 2A**). Upon SEB-stimulation, CD80 and CD86 were similarly expressed on most CD21^pos^ and CD21^low^ B-cell subpopulations and significantly increased expression of both markers was exclusively observed on naïve-like CD21^low^ B cells (**Figure 2B**). This was reflected by low or even significantly reduced further upregulation of the already elevated CD80 and CD86 expression on all CD21^low^ B-cell subpopulations (**Supplementary Figure 2B**). Thus, in the context of SEB-activated co-cultures, only naïve-like CD21^low^ B cells showed still increased expression of CD80 and CD86 compared to the respective CD21^pos^ population, while the induced expression of the co-stimulatory molecules on memory CD21^pos^ and CD21^low^ B cells was comparable.

### Proficient Co-Stimulatory Potential of CD21^low^ B Cell Subsets

To evaluate the co-stimulatory capacity of B cell subpopulations in allogenic co-cultures, the upregulation of activation-associated molecules on third-party CD4 T cells was measured after 24 h. The short culture time was chosen due to limited survival time of CD21^low^ B cells *in vitro* (12, 29). For analysis, the response of naïve and memory T cells was differentiated by gating on CD45RA^pos^ and CD45RA^neg^ T cells.

**Figure 3:**
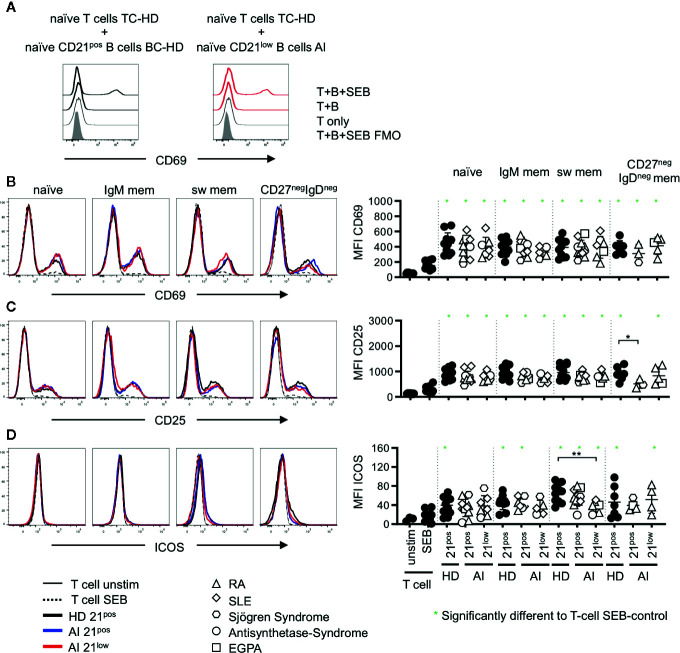
**(A)** Representative histogram overlay displaying CD69 expression in unstimulated T cells (T only) or T-B co-cultures with naïve CD21^pos^ B cells of a HD or naïve CD21^low^ B cells of an AI patients in the absence (T + **(B)** or presence of SEB (T + B + SEB) gated on naïve T cells. **(B**–**D)** Histogram overlays for CD69, CD25 and Inducible Co-stimulator (ICOS) on naïve (CD45RA^pos^) CD4 T cells co-cultured with naïve, IgM mem, sw mem and CD27^neg^IgD^neg^ mem B-cell populations of CD21^pos^ B cells of one representative HD and CD21^pos^ and CD21^low^ populations of one representative patient and statistical analysis thereof. T cells cultured alone and with or without stimulation with SEB were obtained as control. Subsets marked in green (*) were significantly different to the SEB-stimulated T-cell control (p < 0.05). All subsets were statistically significant to unstimulated T cells. Differences were considered significant at *p < 0.05 * and **p < 0.01. The different diseases were marked by different symbols as indicated in the legend.

In unstimulated co-cultures of allogenic T cells and the different isolated B-cell subpopulations, we could not detect a significant upregulation of activation markers as shown for CD69 on naïve (**Figure 3A**) or memory (data not shown) CD4 T cells. In T-B co-cultures with SEB, we observed a significant upregulation of CD69 and CD25 on naïve CD4 T cells when compared to unstimulated or SEB stimulated T-cell cultures without B cells (**Figures 3B, C**), with one exception of CD25-upregulation in co-cultures with CD21^pos^ CD27^neg^IgD^neg^ memory B cells of AI patients. Co-cultures with CD21^low^ B-cell subpopulations induced a comparable upregulation of CD69 and CD25 on naïve CD4 T cells compared to co-cultures with the respective CD21^pos^ population of patients or HD (**Figures 3B, C**). Despite differences in CD86 and CD80 expression between naïve and memory B-cell populations, there were no detectable differences in their capacity to activate T cells, independent of whether CD21^pos^ or CD21^low^ B cells were the APCs. This was also true for the induction of ICOS for most of the CD21^pos^ and CD21^low^ B-cell subpopulations (**Figure 3D**), but not for switched CD21^low^ B cells, which induced significantly less ICOS compared to switched CD21^pos^ B cells of HD (**Figure 3D**).

**Figure 4:**
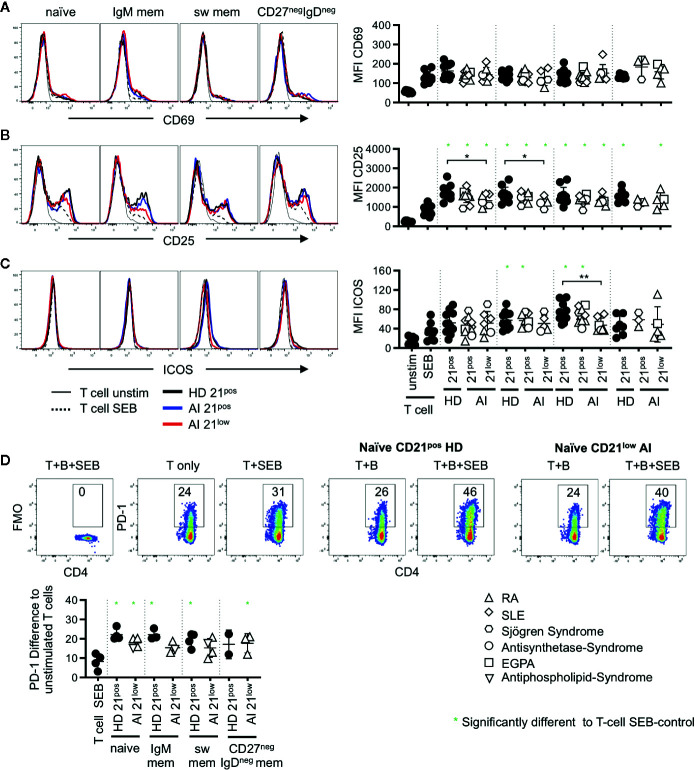
Histogram overlays for CD69 **(A)**, CD25 **(B)**, and ICOS **(C)** expression on memory (CD45RA^neg^) CD4 T cells co-cultured with naïve, IgM mem, sw mem and CD27^neg^IgD^neg^ mem B-cell populations of CD21^pos^ B cells of one representative HD and CD21^pos^ and CD21^low^ populations of one representative patient and statistical analysis thereof. T cells cultured alone and with or without stimulation with SEB were obtained as control. **(D)** Representative FACS plot for CD4 and PD-1 or the FMO in unstimulated T cells, T cells stimulated with SEB and T cells co-cultured with naïve CD21^pos^ B cells of HD or naive CD21^low^ B cells of a patient in the absence or presence of SEB. Graph shows the proportion of PD-1 positive memory T cells in T-cell cultures stimulated as indicated to unstimulated T cells. Subsets marked with green stars were significantly different to the SEB-stimulated T-cell controls. Differences were considered significant at *p < 0.05. The different diseases were marked by different symbols as indicated in the legend. **p < 0.01.

On memory CD4 T cells the induction of CD69 was reduced compared to the naïve CD4 subset (p < 0.01, **Figures 3B**, **4A**) and no significant differences were observed compared to SEB alone. In contrast, the expression of CD25 was higher on memory CD4 T cells after co-cultures with all the different B-cell subpopulations (p < 0.01, **Figures 3C**, **4B**). However unlike for naïve CD4 T cells, the expression of CD25 on memory CD4 T cells was lower in cultures containing naïve-like and IgM memory CD21^low^ B cells of patients compared to co-cultures with the respective CD21^pos^ B cells of HD (**Figure 4B**). This is especially noteworthy since expression of both CD80 and CD86 was higher on naïve-like CD21^low^ B cells in these co-cultures. The expression of ICOS was significantly increased after co-culture of T cells with IgM memory and class switched CD21^pos^ B-cell populations in the presence of SEB compared to T cells stimulated with SEB alone, but no other sorted subpopulation (**Figure 4C**). Similar to the finding in naïve CD4 T cells, the induction of ICOS on memory CD4 T cells by class switched CD21^1ow^ B cells was significantly lower compared to the stimulation by their CD21^pos^ counterpart in HD.

During T-B co-cultures not only co-stimulatory but also inhibitory receptor-ligand pairs are upregulated. We therefore analyzed the expression of PD-1 which was absent after SEB-stimulated co-cultures on naïve CD4 T cells (data not shown) but up-regulated on memory CD4 T cells (**Figure 4D**). In order to determine the activation induced PD-1 expression we subtracted the baseline expression of PD-1 on unstimulated T cells. Significant induction of PD-1 compared to T cells stimulated with SEB alone was observed for all co-cultures with HD-derived CD21^pos^ populations except for IgD^neg^CD27^neg^ B cells, where the number of samples was too low (**Figure 4D**). Similarly, all CD21^low^ B cells induced higher PD-1 expression but due to small sample size only the experiments with naive like CD21^low^ B cells reached significance. Although the experiments could not demonstrate a significant difference in the PD-1 induction between different B-cell populations, there was a trend to a higher induction by naïve CD21^pos^ B cells when compared to their CD21^low^ counterparts as well as to CD21^pos^ switched memory B cells.

T cells response to stimulation by proliferation. Due to the limitations in the co-culture period this could not be addressed by CFSE staining or thymidine incorporation assays and staining for Ki67 was not conclusive (data not shown).

In summary, all CD21^pos^ B cell subsets have similar capacities to induce the investigated activation markers on naïve and memory CD4 T cells with the exception of ICOS induction, which was much stronger after stimulation by switched memory B cells. Interestingly, while T-cell co-stimulation by CD21^low^ subsets was comparable to the respective CD21^pos^ B-cell subsets, switched memory CD21^low^ B-cell subsets did not show the hyper-induction of ICOS.

### Co-Stimulation by CD21^low^ B Cells Does Not Distinctively Bias the T Cell Cytokine Profile

**Figure 5:**
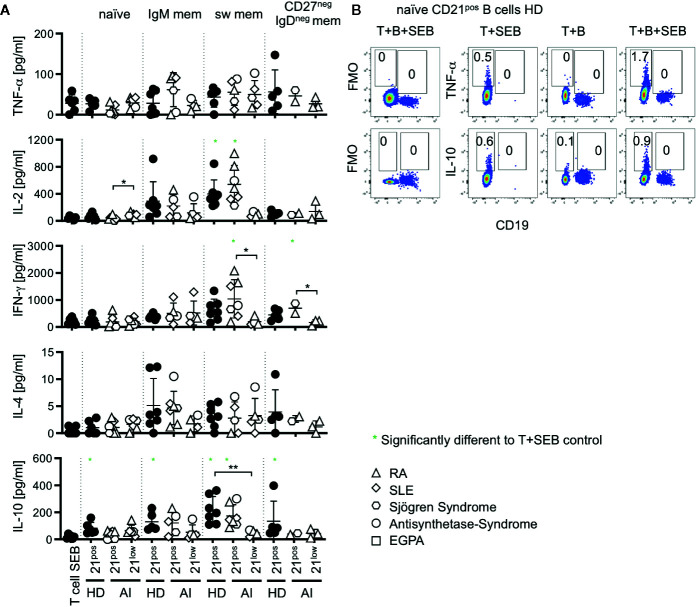
**(A)** Cytokines measured in supernatants of T-B co-cultures. Production of TNF-α, IL-2, IFN-γ, IL-4, and IL-10 was measured in T-B co-cultures with different B-cell subsets of AI patients and HD. Subsets marked in green were significantly different to the SEB-stimulated T-cell control. **(B)** Intracellular cytokine staining of TNF-α and IL-10 versus CD19 in T-B co-cultures with and without SEB and T-cell SEB control. Representative FACS Plots of co-cultures with naïve B cells of HD are displayed. Data are representative for 1–3 times for each CD21^low^ B-cell subpopulation of patients and 2–4 times of each CD21^pos^ B-cell subpopulations of HD in 4 independent experiments. Differences were considered significant at *p < 0.05 and **p < 0.01.

T cells substantially shape the immune response by their cytokine profiles. We addressed if co-cultures with CD21^low^ B cells bias T cells toward a distinct cytokine profile. Thus, IFN-γ, TNF-α, IL-2, IL-4, IL-10, IL-21, IL-17A, and IL-17F production was determined in supernatants of co-cultures after 24 h. Production of IL-17 and IL-21 was not detectable under these conditions (data not shown). The production of TNF-α and IL-4 did not significantly increase after co-cultures with CD21^low^ and CD21^pos^ B-cell subpopulations over the stimulation of T cells with SEB alone (**Figure 5A**). IL-2 production was significantly induced only by co-cultures with switched memory B cells which was, however, not the case for CD21^low^ B cells. For IL-10 all co-cultures with CD21^pos^ B cell-populations of HD were able to induce higher levels than the culture of T cells with SEB alone, reaching the highest levels in cultures with switched memory B cells which was not the case for CD21^low^ B cells, again inducing significantly lower cytokine levels than their CD21^pos^ counterparts. IL-10 was produced by T cells and not B cells in the cultures as demonstrated by flow cytometric analysis (**Figure 5B**). Otherwise we could not detect relevant differences between most CD21^pos^ and CD21^low^ populations, although one has to remark that only few co-cultures reached cytokine levels above the respective SEB-control. In HD-derived samples, switched memory B cells generally induced significantly higher levels of IL-2 (p < 0.001), and IFN-γ (p < 0.01) and tended to higher levels of IL-10 (p = 0.07) than co-cultures with naïve B cells. This pattern was also seen for CD21^pos^ B cells of patients [p < 0.001 (IL-2), p < 0.01 (IFN-γ), and p < 0.01 (IL-10)], but not for CD21^low^ B-cell populations.

The polarization of cultured naïve T cells could not be addressed due to the limited co-culture period. Therefore we analyzed the expansion of the different Th subpopulations according to their CXCR5^pos^ surface expression of CXCR5, CCR6 and CXCR3, allowing the differentiation of Th1, Th1/17, Th17, Th2 cells and circulating T follicular helper cells (cTfh) 1, cTfh1/17, cTfh17 and cTfh2 CD4 T cells (30) (**Supplementary Figure 4A**). In naïve CD4 T cells, non-cTfh cells (CXC5R^neg^CD45RA^neg^) or cTfh cells (CXCR5posCD45RA^neg^) the proportions of CXCR3^pos^CCR6^neg^, CXCR3^pos^CCR6^pos^, CXCR3^neg^CCR6^pos^, or CXCR3^neg^CCR6^neg^ T cells were not significantly altered compared to unstimulated T cells after co-culture with either CD21^low^ B-cell subpopulations of patients or the CD21^pos^ counterparts of HD corroborating the findings of cytokine profiles (**Supplementary Figure 4B**).

In summary, in these short-term co-cultures, we could not detect a clearly altered cytokine profile or preferentially polarized Th-cell expansion when co-cultured with CD21^low^ B cells except switched memory CD21^low^ B cells, which seem to be less efficient to induce the often superior secretion of cytokines by T cells seen in cultures with switched memory CD21^pos^ B cells.

## Discussion

Because of the high base line surface expression of co-stimulatory markers it has been suggested that CD21^low^ B cells are potent APCs, contributing to the immune response by driving the activation and cytokine release of CD4 T cells (20, 31). Thus, we addressed the co-stimulatory capacity of different human CD21^low^ B-cell subsets isolated from peripheral blood of patients with AI diseases by measuring activation of CD4 T cells in allogeneic co-cultures.

We chose SEB-enforced mixed lymphocyte reactions to answer the question about the co-stimulatory potential of distinct CD21^low^ B-cell populations from different autoimmune disorders in order to avoid effects of the heterogeneity of donor T cells and to overcome the lack of specific antigens which renders direct comparison to previously published data (21) not easy.

Despite of the mentioned limitations, our data clearly revealed that primary human CD21^low^ B cells have the potential to serve as APCs: All memory CD21^low^ subsets independent of the underlying disease induced high and—in case of switched memory cells—even significantly higher amounts of HLA-DR when compared to the respective CD21^pos^ counterparts. Interestingly, the expression pattern of HLA-DR on CD21^low^ B cells is reversed to the pattern observed on CD21^pos^ B cells, of which naïve B cells have the highest expression. The high expression of HLA-DR on memory CD21^low^ B-cell subsets is most likely due to exposure to IFN-γ (32, 33) but the cause of the relative reduction in naïve-like CD21^low^ B cells remains less clear. In addition, CD80 and CD86 are highly expressed on all four CD21^low^ B-cell subsets when compared to the respective CD21^pos^ subpopulation of HD *ex vivo* (12, 16–19). This elevated expression was still seen after *in vitro* co-culture on naïve, but not on the different memory CD21^low^ B cells populations, since CD21^low^ memory B cells failed to further upregulate the expression as much as CD21^pos^ memory B cells, as previously reported (6, 13, 24, 28, 29, 34, 35). Other molecules involved in co-stimulation (36–41) were similarly (OX40L) or variably (ICOS-L) expressed between CD21^low^ and CD21^pos^ subpopulations. Also, ICAM-1 known to contribute to co-stimulation by increasing cell-cell interaction (42) was rather increased on all subsets, although the differences were not significant for CD27^pos^ CD21^low^ subsets. On the other hand, CD21^low^ B cells tended also to express higher levels of the inhibitory ligands PD-L1 and PD-L2. Nevertheless, the *ex vivo* phenotype of CD21^low^ B cells marks these cells as activated cells well-equipped for sufficient co-stimulation of T cells at sites of inflammation.

SEB containing T-B co-cultures clearly induced the expression of CD69 and CD25 on naïve and CD25 on memory T cells. CD69 expression on memory T cells was hardly affected by B cells after 24 h, which might be caused by different kinetics compared to naïve T cells. The co-stimulatory effect of the different B-cell subsets, whether naïve or memory, CD21^pos^ or CD21^low^, was nearly identical on naïve T cells regarding CD69 and CD25 induction. Thus, the increased expression of CD80 and CD86 on naïve-like CD21^low^ B cells after co-culture did not translate into a measurably enhanced activation of naïve T cells compared to co-cultures with naïve CD21^pos^ B cells. On the contrary, CD25 induction, which was generally higher on memory than naïve T cells, was even reduced on memory T cells when cultured with naïve and IgM-memory CD21^low^ B cells instead of their CD21^pos^ counterparts. The reason remains elusive. Known signals inducing high CD25 expression as the TCR signal intensity (43) are unlikely to be different in this setting, and IL-2 (44) was even slightly higher in cultures with naïve-like CD21^low^ B cells. Nevertheless, lower CD25 expression may limit the responsiveness of T cells stimulated by interactions with CD21^low^ populations to IL-2 and thereby potentially influence the survival, differentiation, and function of conventional and regulatory T cells (T_reg_) (45, 46) induced in the context of co-stimulation by CD21^low^ B cells *in vivo*. The activation of PD-1^pos^ memory T cells by CD21^low^ B cell populations may additionally be reduced by the generally higher expression of PD-L1 on all four CD21^low^ B-cell subpopulations as reported previously for one of the CD21^low^-like populations (47), given the inhibitory signal transferred by PD-1 ligation (48).

In regard to ICOS expression, we could show for the first time that its expression on naïve and memory T cells was especially high in co-cultures with HD-derived switched-memory B cells when compared to naïve and non-switched memory B cells. Interestingly, this effect was lost for switched memory CD21^low^ B cells. The differential signals given by naïve and switched memory B cells causing the differences in ICOS expression are unknown, but expression of ICOS on T cells is required for the induction and maintenance of an efficient germinal center response and B-cell memory formation (49, 50), implying that switched memory CD21^low^ B cells would be less potent inducers of ICOS-dependent steps during this T-B interaction. In addition, high ICOS expression is required for IL-10, but not the other cytokine production by effector T cells (51). This notion is reflected by our finding that the supernatants of co-cultures with switched-memory B cells of HD contained the highest amount of IL-10, while IL-10 was significantly reduced in the co-culture with CD21^low^ switched memory B cells.

Previous reports in mice suggested an increased antigen presenting potential of age-related B cells (ABCs), the murine homolog of CD21^low^ B cells. *In vitro* and *in vivo* data revealed that ABCs located in the spleen have an enhanced ability to take up, process, and present antigens to T cells compared to follicular B cells resulting in increased activation of T cells as shown by IL-2 secretion and T-cell proliferation (20). We were not able to test antigen-specific APC function of human CD21^low^ B cells of patients with AI disease and comparing results from mice to humans is difficult given the discrepancies of ABCs and CD21^low^ B cells (52) and the different origin of B cells from lymphoid organs in the murine models and peripheral blood in humans (18, 20). Local environment and origin of B-cell subpopulations might strongly contribute to different APC capacities. So far, only few and conflicting studies have been published addressing the co-stimulatory potential of CD21^low^ B cells in humans. In contrast to our findings, Shimabukuro-Vornhagen and colleagues described a CD21^low^CD86^high^ B-cell population of HD to induce increased proliferation and upregulation of CD25 on autologous T cells in the presence of anti-CD3 when compared to CD21^pos^CD86^low^ B cells (21). Comparing, however, the CD21^low^CD86^high^ and CD21^pos^CD86^high^ B-cell populations, the APC capacity was not significantly increased, which is consistent with our observation. Besides differences in experimental settings, as they looked at an autologous co-culture setting, co-stimulated with anti-CD3 instead of SEB and observed a culture time of seven days, the B-cell subsets were obtained from HD and contained an unidentified mixture of B-cell subpopulations, which impedes the comparison. On the other hand, very recently reduced co-stimulatory potential was shown for a CD20^high^CD24^low/-^ atypical memory B-cell population of SLE patients, largely corresponding to CD21^low^ B cells, compared to CD27^pos^ memory B cells (53). The reduced APC-capacity of total CD21^low^ B cells was shown in a setting with low dose CD3 stimulation after five days. Limited survival of CD21^low^ B cells *in vitro* due to increased apoptosis (12, 15, 17, 29, 53) may, however, strongly influence the results in all settings with incubation times of more than 2 days.

Based on high T-bet expression and the preferential usage of IgG3 isotypes in CD21^low^ B cells (32, 54–56), the formation of CD21^low^ B cells has been linked to Th1 responses and IFN-γ (54, 57, 58). Whether CD21^low^ B cells can vice-versa shape T-cell differentiation toward a Th1 profile is not clear. In mice, Hao et al. had reported an increased potential of ABCs to polarize naïve CD4 T cells toward a Th17 profile when performed under Th17 polarizing condition (31). Under Th1 and T_reg_ polarizing conditions, no difference was observed between the co-culture with follicular B cells and ABCs. Due to the limited survival of CD21^low^ B cells *in vitro*, we performed short-term cultures, which don’t allow for effector polarization from unprimed T cells. Acknowledging these limitations of the used *in vitro* conditions, we did not detect clear changes in the composition of naïve, non-Tfh and Tfh memory T cells according to their chemokine receptor profiles.

Accordingly, our data demonstrated no significant alterations for most cytokine concentrations in the supernatants of co-cultures with CD21^low^ B cells compared to CD21^pos^ subsets with a few exceptions. Compatible to data of a murine model (20), the co-culture with naïve CD21^low^ B cells induced higher IL-2 levels when compared to naïve CD21^pos^ B cells, although much less than co-cultures with CD21^pos^ memory B cells. More importantly, however, the superior co-stimulatory capacity of switched memory CD21^pos^ B cells compared to all other CD21^pos^ B-cell subsets, which we could observe for ICOS induction and secretion of IL-2, IFN-γ, and IL-10, is in line with previous observations (3) but was not observed for the respective CD21^low^ B-cell population, which was just within the range of the other B-cell subsets.

Given the well-equipped function as potential APCs, CD21^low^ B cells might present potential targets for therapeutic interventions in AI patients, since the high expression of co-stimulatory molecules on these cells is combined with an increased incidence of auto-reactive BCR entities among CD21^low^ B cells in RA, CVID, and SLE (12, 14, 17). The impact of CD80 and CD86 mediated co-stimulation on disease activity of AI diseases was emphasized by the efficacy of CD80/CD86 inhibition (59, 60). Abatacept, a fusion protein directly binding to CD80/CD86, approved for second line therapy in patients with RA, has been shown to directly decrease the expression of both receptors on B cells, resulting in reduced co-stimulation of T cells (61). How much of this *in vivo* effect is owed to a direct effect on the CD21^low^ B-cell compartment remains elusive. Nevertheless, either the elimination or the inhibition of the co-stimulatory potential of CD21^low^ B cells appears reasonable therapeutic approaches in patients with AI disorder and expansion of this B-cell population. On the other hand, the co-stimulatory potential of CD21^low^ B cells seems to be important for an efficient immune response against influenza (62). Further studies are currently conducted to address this issue and to elucidate the impact of Abatacept in other disease conditions linked to the occurrence of potentially autoreactive CD21^low^ B cells.

## Data Availability Statement

All datasets generated for this study are included in the article/**Supplementary Material**.

## Ethics Statement

The studies involving human participants were reviewed and approved by Freiburg 239/1999 and 121/11 and Freiburg 66/13. The patients/participants provided their written informed consent to participate in this study.

## Author Contributions

MR performed the experiments, analyzed and interpreted the data, and wrote the manuscript. KP, VS, and IH performed experiments. RV contributed to the acquisition and sample collection of patients and critically revised the manuscript. KW and BK designed and coordinated the study, interpreted data and wrote the manuscript. All authors contributed to the article and approved the submitted version.

## Funding

This work was supported by the Deutsche Forschungsgemeinschaft (grant TRR130; P07 to KW and P12 to RV) and the German Federal Ministry of Education and Research (grant BMBF 01E01303).

## Conflict of Interest

The authors declare that the research was conducted in the absence of any commercial or financial relationships that could be construed as a potential conflict of interest.

## Abbreviations

ABCs, Age-related B cells; AI, autoimmune; APCs, Antigen-presenting cells; BCR, B-cell receptor; cTfh, circulating T follicular helper cells; CVID, Common variable immunodeficiency; CRP, C-reactive protein; DAS-28, Disease activity score 28; EGPA, Eosinophilic granulomatosis with polyangiitis; HCV, Hepatitis C virus; HD, Healthy donor; HIV, Human immunodeficiency virus; ICOS, Inducible Co-stimulator; MACS, Magnetic cell-sorting; PBMCs, Peripheral blood mononuclear cells; PD, programmed cell death; pSS, primary Sjögren Syndrome; RA, Rheumatoid arthritis; SD, Standard deviation; SEB, *Staphylococcus* enterotoxin B; SLE, Systemic lupus erythematosus; SLEDAI, Systemic lupus erythematosus disease activity index; SSc, Systemic Sclerosis; T-B, T cell – B cell; T_reg,_ Regulatory T cell.
