# Supplementary material for: The Antigen Presenting Potential of CD21low B Cells
Source: Front Immunol. 2020 Oct 21;11:535784. doi: 10.3389/fimmu.2020.535784 (PMC7609862; doi:10.3389/fimmu.2020.535784)
Supplement: Supplementary file 1 [file DataSheet_1.pdf]

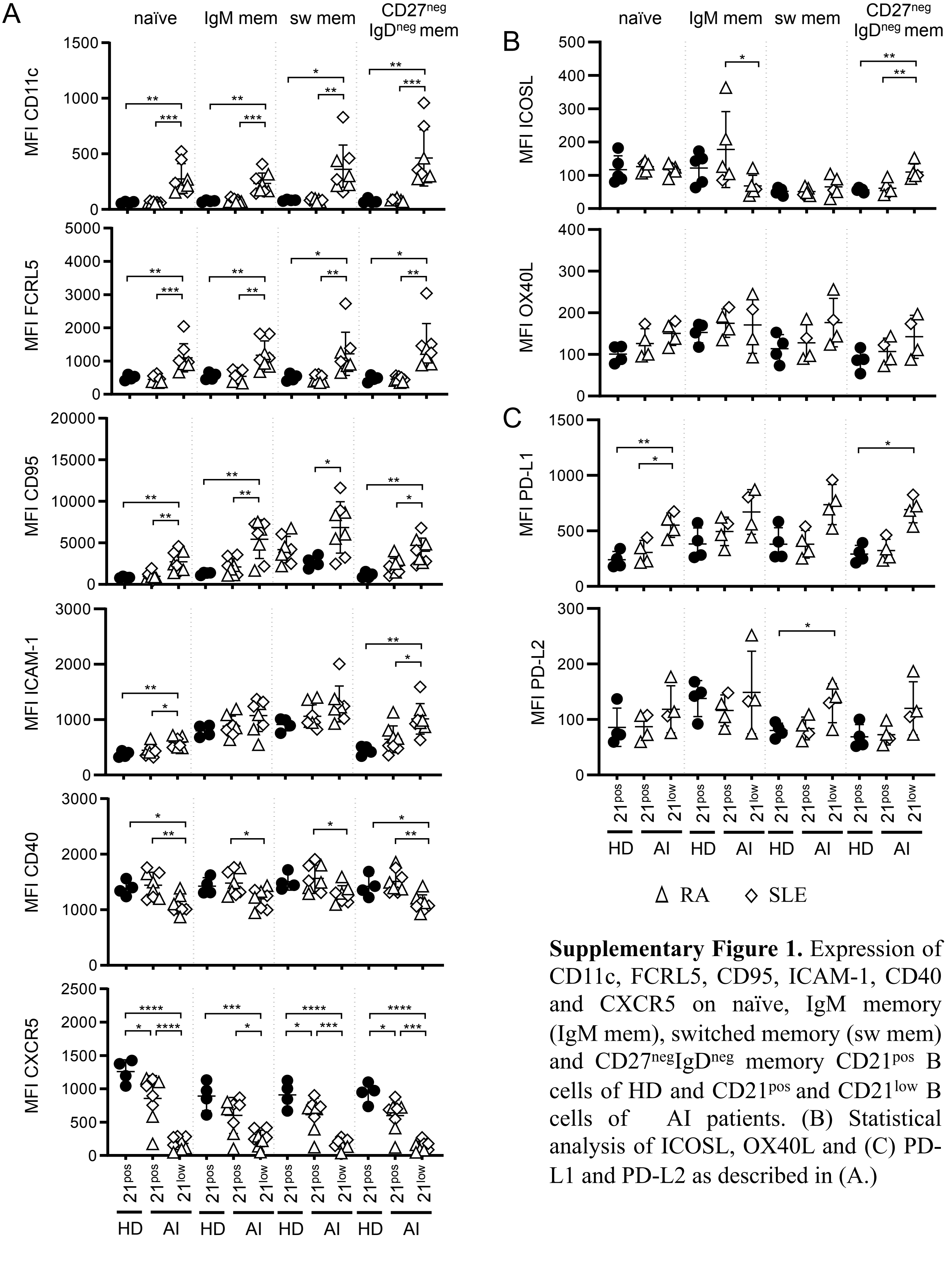

Supplementary Figure 1. Expression of CD11c, FCRL5, CD95, ICAM-1, CD40 and CXCR5 on naïve, IgM memory (IgM mem), switched memory (sw mem) and CD27[neg]IgD[neg] memory CD21[pos] B cells of HD and CD21[pos] and CD21[low] B cells of AI patients. (B) Statistical analysis of ICOSL, OX40L and (C) PD-L1 and PD-L2 as described in (A.)

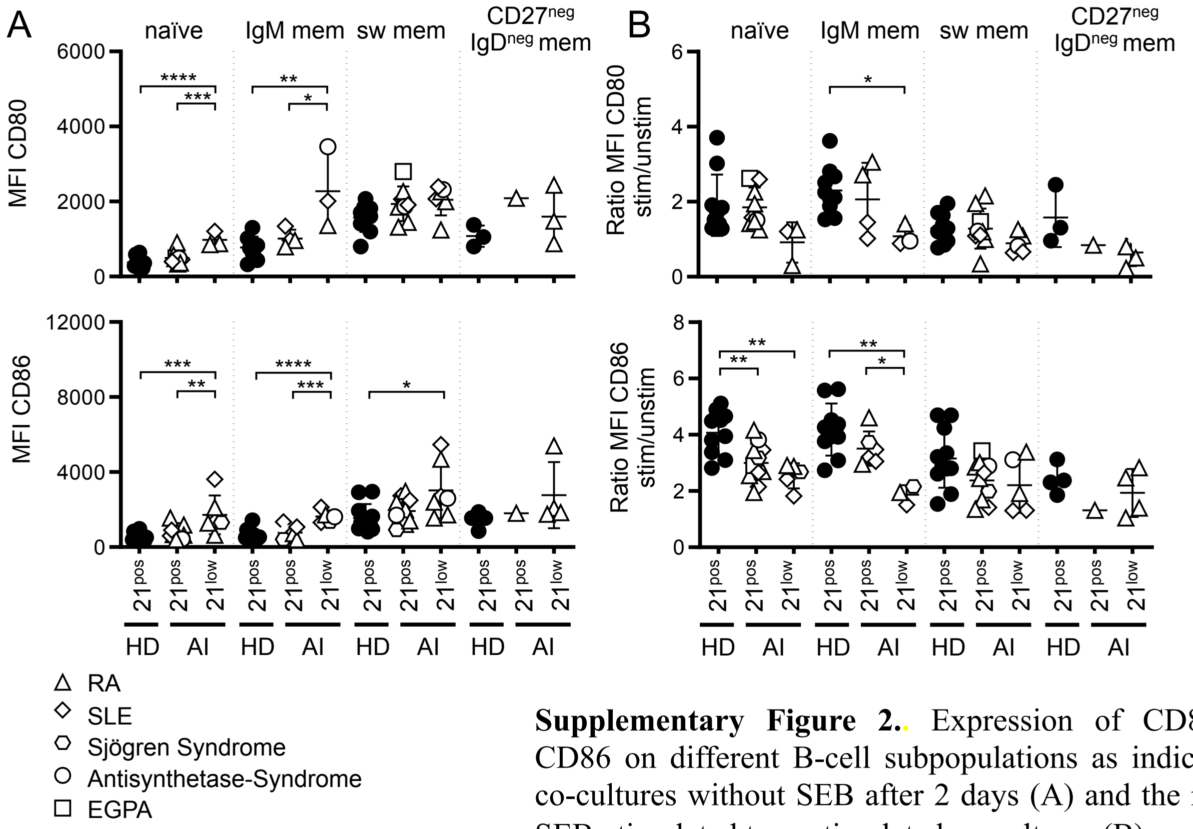

Supplementary Figure 2. Expression of CD80 and CD86 on different B-cell subpopulations as indicated in co-cultures without SEB after 2 days (A) and the ratio of SEB-stimulated to unstimulated co-cultures (B).

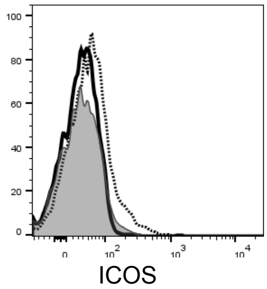

Supplementary Figure 3: Representative FACS plot for ICOS gated on CD45R0 T cells. Histograms show T cells cultured alone (black line) or T cells from T-B co-culture stimulated with CD27^{neg}IgD^{neg} memory B cells of HD in the presence of SEB (dashed black line).The tinted grey line show ICOS expression on CD27^{neg}IgD^{neg} memory B cells of the same donor as a negative control.

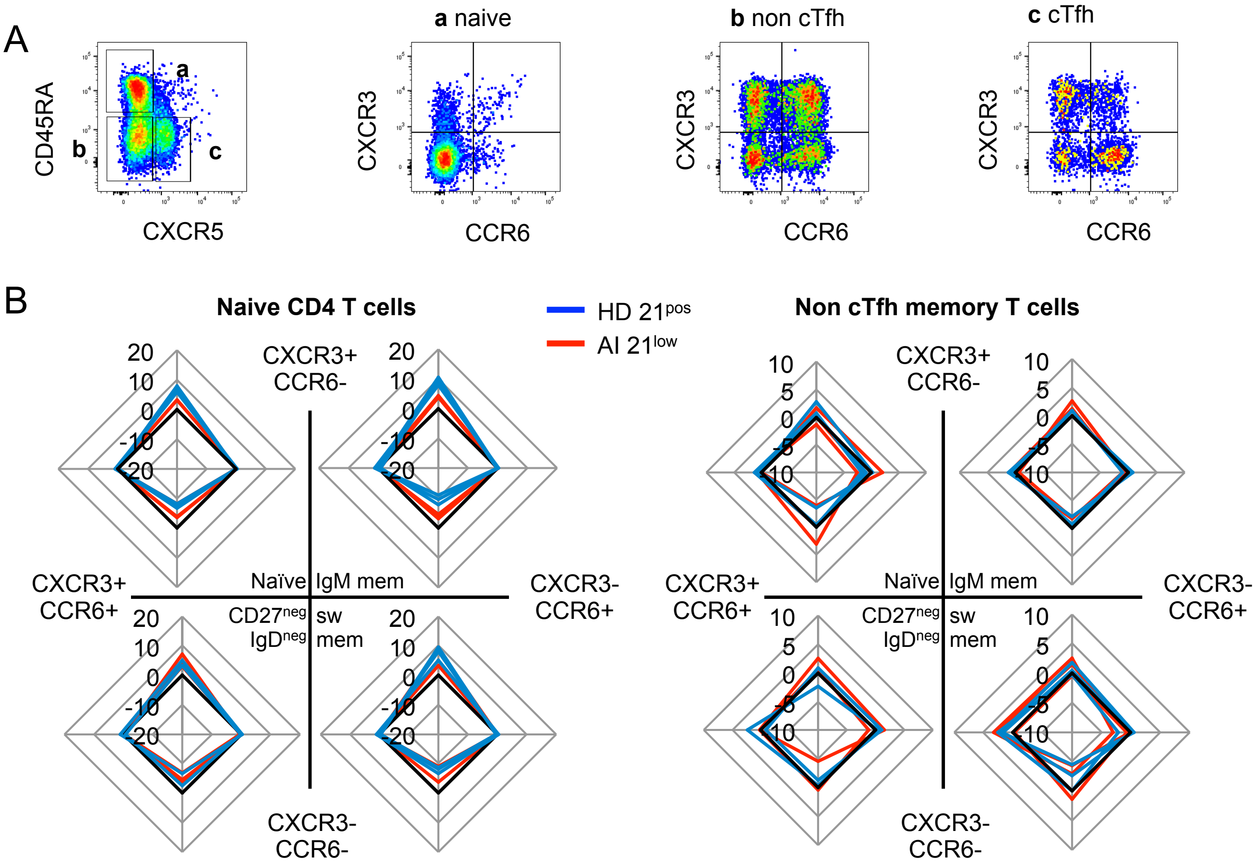

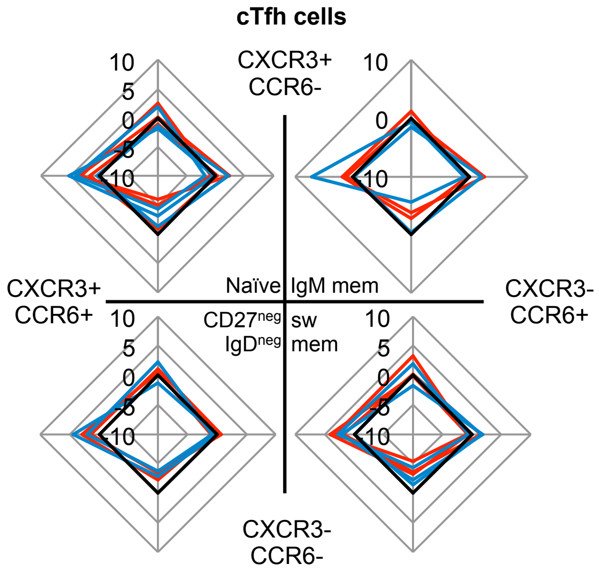

Supplementary Figure 4 (A) Gating strategy and representative FACS-plots for the differentiation of (a) naïve (CD45RA^{pos}CXCR5^{neg}), (b) non cTfh memory (CD45RA^{pos}CXCR5^{neg}) and (c) cTfh cells (CD45RA^{neg}CXCR5^{pos}) and the determination of CXCR3^{pos}CCR6^{neg}, CXCR3^{pos}CCR6^{pos}, CXCR3^{neg}CCR6^{pos} or CXCR3^{neg}CCR6^{neg} cells in unstimulated T cell. (B) Radar plots show the polarization of naïve, cTfh memory and non-cTfh memory T cells as seen by changes in the proportions of CXCR3^{pos}CCR6^{neg}, CXCR3^{pos}CCR6^{pos}, CXCR3^{neg}CCR6^{pos} or CXCR3^{neg}CCR6^{neg} cells in the respective T-cell subpopulation. The pictured T cells were co-cultured with naïve CD21^{pos} B cells of HD or naïve CD21^{low} B cells of AI patients. Graphs are showing the difference of T cell subsets in co-culture with SEB to T cells stimulated with SEB alone in three independent experiments.

Supplementary Table 1

				**HD CD21^{pos}**				**Pat CD21^{pos}**				**Pat CD21l^{ow}**					
**Assay**	**patient #**	**B cell HD #**	**T cell HD #**	naive	IgM mem	sw mem	CD27^{neg}IgD^{neg} mem	naive	IgM mem	sw mem	CD27^{neg}IgD^{neg} mem	naive	IgM mem	sw mem	CD27^{neg}IgD^{neg} mem	**Surface staining**	**Cytokines**
#1	#1	#1	#12	x	x	x		x	x	x					x	x	
#2	#2	#2	#13	x	x	x										x	x
#3	#3	#3	#14	x	x	x		x		x		x			x	x	x
#4	#4	#4	#15	x	x	x	x	x	x	x	x	x	x			x	x
	#5	#4	#15					x	x	x		x	x			x	x
#5	No patient	#5	#16	x	x	x	x									x	
#6	#6	#6	#17	x	x	x		x	x	x	x			x		x	
	#7	#6	#17					x	x	x				x	x	x	
#7	#8	#7	#18	x	x	x	x	x		x	x		x	x	x	x	x
#8	#9	#8	#19	x	x	x	x	x	x	x		x	x	x		x	
#9	#10	#9	#20	x	x	x	x	x	x	x		x	x	x		x	x
#10	#11	#10	#21	x	x	x	x	x	x	x		x		x	x	x	x
#11	#12	#11	#22	x	x	x	x	x	x	x		x	x	x		x	
	**Total:**			11	11	11	7	11	9	11	3	7	6	7	5	13	7
